# Concealed Inherited Cardiomyopathies Detected in Cardio-Oncology Screening

**DOI:** 10.3390/jcm13010002

**Published:** 2023-12-19

**Authors:** Rebeca Lorca, Isaac Pascual, Maria Fernandez, Rut Alvarez-Velasco, Santiago Colunga, Maria Muñiz, Marta Izquierdo, Yolanda Fernandez, Emilio Esteban, Juan Gomez, Pablo Avanzas, Teresa Lopez-Fernandez

**Affiliations:** 1 Área del Corazón, Hospital Universitario Central Asturias, 33011 Oviedo, Spain; pascualisaac@uniovi.es (I.P.); mariafndz27@gmail.com (M.F.); rutalvarez3@gmail.com (R.A.-V.); santicolunga@hotmail.com (S.C.); avanzas@secardiologia.es (P.A.); 2 Instituto de Investigación Sanitaria del Principado de Asturias (ISPA), 33011 Oviedo, Spain; juan.gomezde@sespa.es; 3 Departamento de Biología Funcional. Área de Fisiología, Universidad de Oviedo, 33003 Oviedo, Spain; 4 Unidad de Cardiopatías Familiares, Área del Corazón y Departamento de Genética Molecular, Hospital Universitario Central Asturias, 33011 Oviedo, Spain; 5 Redes de Investigación Cooperativa Orientadas a Resultados en Salud (RICORs), 28029 Madrid, Spain; 6 Departamento de Medicina, Universidad de Oviedo, 33003 Oviedo, Spain; eestebang@seom.org; 7 Oncología Médica, Hospital Universitario Central Asturias, 33011 Oviedo, Spain; mmunizcastillo@gmail.com (M.M.); izqman@gmail.com (M.I.); yolandafp2004@yahoo.es (Y.F.); 8 Centro de Investigación en Red de Enfermedades Cardiovasculares (CIBERCV), 28029 Madrid, Spain; 9 Cardiología, Hospital Universitario la Paz, IdiPAZ Research Institute, 28046 Madrid, Spain; tlfernandez8@gmail.com

**Keywords:** cardio-oncology, cardiomyopathy, genetics

## Abstract

Introduction: Basal cardiovascular risk assessment in cardio-oncology is essential. Integrating clinical information, ECG and transthoracic echocardiogram can identify concealed inherited cardiomyopathies (ICMPs) with potential added risk of cardiotoxicity. We aimed to evaluate the impact of our Cardio-Oncology Unit design in detecting concealed ICMPs. Methods: We carried out a retrospective study of all consecutive breast cancer patients referred to the Cardio-Oncology Unit for cardiac evaluation (2020–2022). ICMPs diagnosis was provided according to ESC guidelines and underwent genetic testing. ICMPs prevalence in this cohort was compared to the highest and lowest frequency reported in the general population. Results: Among 591 breast cancer patients, we identified eight patients with ICMPs: one arrhythmogenic cardiomyopathy (ACM), three familial non-ischemic dilated cardiomyopathy (DCM), three hypertrophic cardiomyopathy (HCM) and one left ventricular non-compaction cardiomyopathy (LVNC), which has now been reclassified as non-dilated left ventricular cardiomyopathy. The number of ICMPs identified was within the expected range (neither overdiagnosed nor overlooked): ACM 0.0017 vs. 0.0002–0.001 (*p* 0.01–0.593); DCM 0.0051 vs. 0.002–0.0051 (*p* 0.094–0.676); HCM 0.005 vs. 0.0002–0.002 (*p* < 0.001–0.099); LVCN 0.0017 vs. 0.00014–0.013 (*p* 0.011–0.015). Genetic testing identified a pathogenic *FLNC* variant and two pathogenic *TTN* variants. Conclusion: Opportunistic screening of ICMPs during basal cardiovascular risk assessment can identify high-risk cancer patients who benefit from personalized medicine and enables extension of prevention strategies to all available relatives at concealed high cardiovascular risk.

## 1. Introduction

Over the past few decades, given encouraging improvement in cancer-related mortality [1], cardiovascular (CV) disease (CVD) and complications have become a major concern in cancer patients. Some very useful known cancer treatments, including anthracycline chemotherapy (AC) and anti-HER2 targeted therapies, have been associated with induced cardiomyopathy, left ventricular ejection fraction (LVEF) dysfunction and heart failure (HF) [2,3]. Apart from that, the number of cancer patients who already have pre-existing CVD or CV risk factors (CVRF) is also increasing. As a result, cancer treatments with potential CV toxicity require specific cardiac surveillance protocols. In this challenging scenario, cardio-oncology has emerged as a new discipline to improve the management of patients with both cancer and CVD [2,3,4,5].

Cardio-oncology multidisciplinary teams arise with the main goal of helping the management of cancer patients, planning their best possible cancer treatments while trying to safely minimize their CV complications [5]. In this regard, in 2022, the European Society of Cardiology (ESC) published the very first guidelines on cardio-oncology [3], a major breakthrough in the field. Accordantly, all cancer patients should undergo a baseline CV risk stratification before starting any potentially cardiotoxic anticancer therapy [2,4,5,6,7,8,9] (class I, level B [3]). This baseline evaluation should include, at least, personal history of pre-existing CVD or previous cardiotoxic cancer treatment and CVRF assessment (including smoking, diabetes mellitus (DM), hypertension (HTN) or dyslipidemia (DL), lifestyle, etc.) [5]. Moreover, basal electrocardiogram (ECG) is recommended (class I, level C) in all patients [3]. Further choice of additional CV tests should be individualized [3]. In this sense, cardiac imaging plays a central role in cardiotoxicity risk evaluation of many patients [10]. For instance, baseline transthoracic echocardiography (TTE) is recommended in all patients before anti-HER2 or AC (class I, level B) [5,11,12,13,14,15].

However, in the era of precision medicine, we believe that basal CV assessment should go beyond classical CVRF and LVEF evaluation [10]. In this regard, identifying inherited cardiomyopathies (ICMPs) in CV basal screening could be of utmost importance [16]. In fact, recent 2023 ESC guidelines encourage clinicians to use a ‘cardiomyopathy mindset’ to identify these patients with genetic cardiomyopathies at every clinical phase, from subclinical (or concealed) to overt and/or at end stage [17].

In this study, we aimed to evaluate the impact of integral CV assessment at our Cardio-Oncology Unit in detecting concealed ICMPs. Our Cardio-Oncology Unit was designed in the COVID-19 pandemic scenario to minimize hospital visits [18]. As a result, all cancer patients who needed basal TTE underwent on the same first visit an integral basal CV assessment including global clinical evaluation plus CV risk stratification, ECG and TTE [18].

## 2. Materials and Methods

### 2.1. Patients

In this retrospective study, we reviewed all consecutive breast cancer patients who were referred to the Cardio-Oncology Unit for cardiac evaluation during three consecutive years (2020–2022). The study was conducted in accordance with the Declaration of Helsinki and approved by the Institutional Ethics Committee (CEImPA 2023.264).

**Figure 1:**
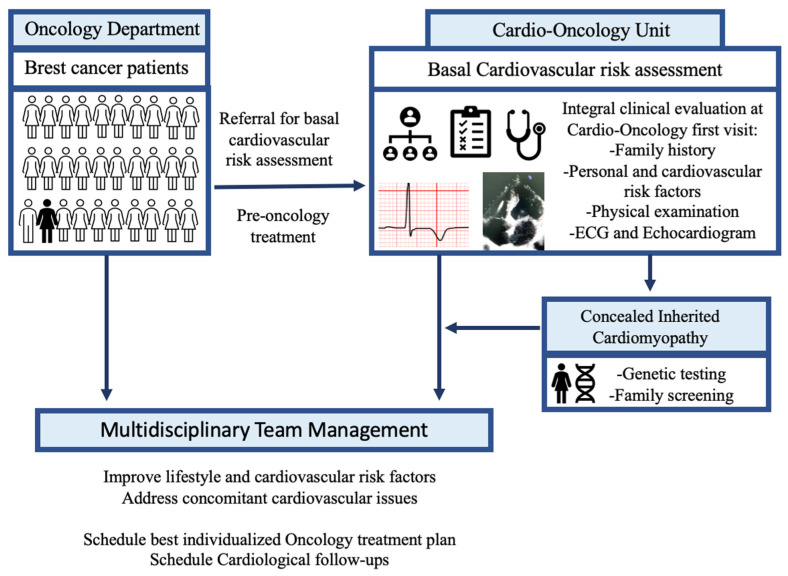
**Clinical diagnostic workflow.** All breast cancer patients are referred for basal cardiovascular risk assessment. Thanks to integral clinical evaluation at Cardio-Oncology Unit, patients with concealed inherited cardiomyopathies can be identified. All patients undergo multidisciplinary team management. ECG, electrocardiogram.

Their first cardiological evaluation at the Cardio-Oncology Unit was performed by the same cardiologist, a specialist in cardio-oncology and ICMPs. Basal integral CV assessment at the Cardio-Oncology Unit included: (1) basal ECG; (2) history and physical examination: review of clinical history (both family and personal history, including planned and prior oncological treatment), anamnesis and physical examination; (3) TTE (Figure 1). All cardiological visits took place in the same clinical practice with the same electrocardiograph and echocardiogram (Philips—Affinity 50 Ultrasound System). This study was approved by the local Institutional Ethical Committee (CEImPA 2023.264).

We identified those cancer patients with a new ICMPs final diagnosis at cardio-oncology evaluation (Figure 1): hypertrophic cardiomyopathy (HCM), familial non-ischemic dilated cardiomyopathy (DCM) or arrhythmogenic cardiomyopathy (ACM), according to ESC guidelines [6,19,20]. Borderline phenotypes, or those who presented alternative explaining conditions [6,17,19,20], were not included. Only patients previously labeled as left ventricular non-compaction cardiomyopathy (LVNC) who met current criteria for non-dilated left ventricular cardiomyopathy (NDLVC) were reclassified as NDLVC [17]. Although, for the purpose of this study, patients with arrhythmogenic cardiomyopathy are presented as so, some of the patients with “left ACM” would now be reclassified as NDLVC or DCM [17].

Cardiological follow-up, evaluation and treatment, including implantable cardioverter device (ICD) implantations, were also performed according to ESC guidelines [6,19,20]. Clinical data from ECG, TTE and cardiac magnetic resonance (CMR), when available, as well as medical treatment, gender, age, oncological treatment and CVRF, including HTN, smoking history, DM, DL and the presence of familial or personal history for premature CVD or sudden cardiac death (SCD), were collected.

### 2.2. Genetic Study and Variant Classification

Genetic testing was offered according to guidelines [6,19,21]. All patients who agreed to participate signed the informed consent for genetic testing (local Institutional Ethical Committee approval, CEImPA 2022.254). DNA was obtained from peripheral blood, and NGS analysis was performed, as reported elsewhere [22,23,24,25,26]. The NGS cardiovascular panel provided in our institution was designed with the aim of optimizing economic resources, attempting to be both time effective and cost effective. Therefore, our last version of the NGS cardiovascular panel [27] analyzes more than 200 genes and includes all minimum genes related to inherited cardiac conditions [6,19,21]. As a result, all patients with inherited cardiovascular conditions undergo the same NGS cardiovascular panel, irrespective of the cardiovascular phenotype. Complete information about the NGS cardiovascular panel can be consulted in Supplementary Table S1.

Interpretation of genetic variants with an allele frequency < 0.01 was based on the American College of Medical Genetics and Genomics (ACMG-AMP) 2015 Standards and Guidelines criteria [28]. Selected variants were evaluated by a cardiologist and a biologist specialized in inherited CV conditions. According to ACMG criteria, all variants were considered as pathogenic/likely pathogenic (P/LP), variants of uncertain significance (VUS) or benign/likely benign. All genetic rare variants identified in those genes with definitive or strong evidence for pathogenicity for each inherited cardiac condition were evaluated and classified according to ACM Criteria [28], initially regardless of the patient’s phenotype. Sanger sequencing of the corresponding PCR fragments was used to confirm the presence of VUS, LP and P variants. Afterwards, to achieve the final classification of VUS, LP and P variants, clinical data of all carriers were carefully reviewed before concluding with the final genetic report. Incidental or secondary findings (results that are not related to the indication for ordering the sequencing but that may nonetheless be of medical value or utility to the ordering physician and the patient) were reported according to ACMG recommendations. Finally, in this study, only P/LP variants associated with the cardiological phenotype are reported in the results section.

### 2.3. Statistical Analysis

Statistical analyses were performed with SPSS v.19. Descriptive data for continuous variables are presented as mean ± SD and as frequencies or percentages for categorical variables. ICMPs prevalence in this cohort was compared to the highest and lowest frequency reported in the general population. DCM has an estimated prevalence of 1 in 250–500; HCM ranges between 1 in 500 and 1 in 5000 and ACM between 1 in 1000 and 1 in 5000 persons [16,19]. Real prevalence of LVNC is unknown. Reported prevalence varies from 0.014 to 1.3% [29,30,31,32,33,34,35]. Differences frequencies were evaluated with the Chi-square test, and continuous variables were compared with the Student’s *t*-test. Differences were considered to be significant if the *p* value was below 0.05.

## 3. Results

From 2020 to 2022, 591 breast cancer patients were referred to the Cardio-Oncology Unit for cardiological evaluation. Seven patients had a prior history of LVEF deterioration under oncology treatment before 2020, and another two were referred with a severe reduced LVEF due to prior ischemic coronary artery disease.

Mean age at the first Cardio-Oncology visit was 57.5 (±12.9 SD), and most breast cancer patients were women (99.5%). General clinical characteristics of the cohort are shown in Table 1.

**Figure 2:**
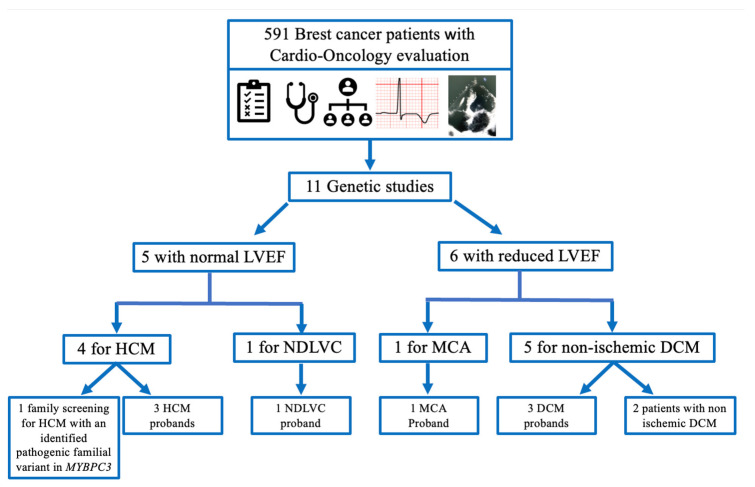
**Flowchart of breast cancer patients with genetic testing.** Cardio-oncology evaluation (cardiovascular risk stratification including cardiotoxic cancer treatment, physical examination, family history of cardiovascular disease).

Based on cardio-oncology evaluation, genetic testing was indicated in 11 patients (Figure 2), 5 of them with normal LVEF. For instance, genetic screening was indicated in one patient due to family history of HCM. Sanger sequencing revealed that she was not carrier of the familial pathogenic *MYBPC* G263*. Four other patients were sequenced due to HCM diagnosis or NDLVC (with fibrosis and hypertrabeculation on CRM, previously classified as LVNC).

**Figure 3:**
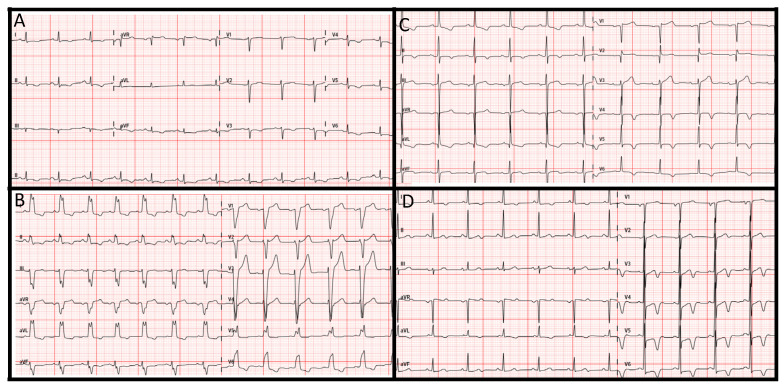
**Electrocardiograms**. (**A**) Patient 1 with arrhythmogenic cardiomyopathy; (**B**) patient 4 with dilated cardiomyopathy; (**C**) patient 6 with hypertrophic cardiomyopathy and apical aneurism; (**D**) patient 7 with apical hypertrophic cardiomyopathy.

On the other hand, the other six patients who underwent genetic testing did present reduced LVEF. Clinical characteristics and genetic findings are shown in Table 2. Suspicion of ICMP was based on ECG findings (Figure 3) and family history, considering cardiotoxic cancer treatment. For instance, patient 1 was one of the seven patients with an LVEF deterioration under oncology treatment before 2020. When she was referred to the new Cardio-Oncology Unit in 2020, her last LVEF was 48%. Despite it was thought that the decrease in LVEF was secondary to anthracycline chemotherapy; her ECG with negative T waves at inferior leads, I and from v3 to v6, suggested otherwise (Figure 3A). CMR confirmed ICMP suspicion, and genetic testing revealed she was carrier of the pathogenic truncating variant in *FLNC* p.Tyr1042Ter, leading to the final diagnosis of left ACM (now it should also be reclassified as NDLVC). As none of her parents was carriers of the variant, it was considered de novo.

At basal evaluation, only one patient (patient 4, Table 2) presented concealed DCM, with asymptomatic severely reduced LVEF and abnormal ECG (Figure 3B). From the 22 patients whose LVEF deteriorated during follow-up, only 5 patients (22.7%) were considered for genetic testing due clinical suspicion of ICMP. Genetic testing identified an underlying cause in three of these five cases (60%) (Table 2). In the absence of other supporting data, the two patients with ICMP suspicion but negative genetic results were not considered as conclusively having ICMP (only “non-ischemic” DCM), and, subsequently, are not presented as so in this study. However, further information about clinical family screening could provide more insights about their disease.

Thus, we performed a new diagnosis of ICMP in eight patients (one ACM, three DCM, three HCM and one NDLVC with hypertrabeculation) and could rule out one familial HCM. The number of ICMPs identified in the 591 consecutive breast cancer patients was a bit higher considering the lowest frequencies reported in a random population but within the expected range of the highest reported ones [16,19,29,30,31,32,33,34,35] (Table 3).

SCD risk stratification was carefully evaluated in all patients diagnosed with ICMP and their relatives. Cancer treatment was scheduled with close cardiological follow-up, and all patients successfully completed planned cancer treatment. In all HCM patients, LVEF remained normal.

## 4. Discussion

The frequency of ICMPs in a cancer population should be the same as in the general healthy population. Consequently, in our cohort, thanks to the integral cardiovascular basal evaluation performed at the Cardio-Oncology Unit, we were able to identify the expected numbers of ICMPs (Table 3) that otherwise may have been overlooked. This means that ICMPs were neither overdiagnosed nor overlooked in this cancer cohort.

To date, very few studies have evaluated the prevalence of ICMP at baseline CV assessment in cancer populations. Most studies have focused on evaluating the role of genetic predisposition in those patients who develop HF or LVEF dysfunction during/after cancer treatment. For instance, pathogenic variants and rare variants in *TTN* and *MYH7* genes have been reported in patients with chemotherapy-induced cardiomyopathy [36,37,38,39,40,41]. However, to the best of our knowledge, this is the first study to evaluate the impact of cardio-oncology CV basal screening in detection of ICMPs. In this regard, we would like to reinforce the importance of CV basal risk evaluation, including ECG and family history investigation.

The emergence of the cardio-oncology discipline and recent ESC guidelines have provided important insights that have substantially helped improve cancer patients management in many challenging scenarios [2,3,4]. The previous cardiotoxicity surveillance of many studies and oncology trials during anticancer therapies just focused on LVEF measurements. Therefore, some physicians may only focus their cardiotoxicity awareness on evaluation of this single parameter [10]. However, TTE has an important role beyond LVEF changes recognition. TTE can also reveal subclinical unknown CVD [5,10,42], including ICMPs. Moreover, basal CV risk stratification, including ECG, is also essential and recommended in all cancer patients [3]. In this regard, the CARDIOTOX registry [9] is one of the most important studies that has illustrated the importance of baseline CV risk assessment. Basal SCORE [43] results could predict severe cardiotoxicity and all-cause mortality during follow-up [9].

In the present ESC guidelines, the recommendation for cardiological referral is limited to those patients whose baseline CV toxicity risk assessment presents abnormal findings, those who have pre-existing CVD or ECG abnormalities or those who are at high or very high risk (class I, level C) [3]. Although not explicitly taken into account in the scores, excluding family history of cardiomyopathy and ruling out that the patient is carrier of a pathogenic gene variant associated with a known familiar cardiomyopathy is also required to consider the patient as “low risk” [3,9,10,43]. In this regard, planning protocols for cancer treatments with potential CV toxicity provides unique opportunities to comprehensively assess CV health before initiation of cancer treatment [5]. However, basal risk assessment can be challenging. Our Cardio-Oncology Unit began in 2020, coinciding with the rise of the COVID-19 pandemic [18]. Therefore, to minimize hospital visits, we took advantage of the possibility to integrate the TTE evaluation with a general cardiac evaluation, including personal and family history, physical examination, anamnesis and global CV risk assessment, including ECG, at the same visit. As a result, considering that all breast cancer patients scheduled for AC or anti-HER therapies will need a pre-treatment basal TTE [3], we specifically designed a breast cancer patient flow chart. The primary objective was to avoid waiting lists to prevent cancer treatment delays in the pandemic scenario.

Given the presented data, we encourage clinicians to invest a little more time to gather information about family history of suspected ICMP and SCD at a young age and carefully look for ECG abnormalities. Moreover, we also hypothesize that, in those patients whose TTE is mandatory, scheduling a basal cardiology consult in the same visit, including both TTE plus clinical evaluation and CV risk assessment (including family history and ECG), could be of value and, perhaps, cost effective.

On the other hand, identifying concealed ICMPs in cancer patients is of utmost importance as they can represent a potentially very-high-risk population for cardiotoxicity. In our cohort, cancer treatment was discussed by the Cardio-Oncology team to plan the best possible cancer treatment while trying to safely minimize its CV complications. Multidisciplinary work, properly scheduling cardiological and cancer treatment, was the goal so that all our patients could successfully complete their planned treatment. In this regard, in patient 4, ICD-TRC implantation was delayed until radiotherapy and optimal medical treatment were completed. Afterwards, both LVEF and the severe mitral regurgitation, as well as HF symptoms, significantly improved. In addition, genetic data can provide very valuable information for personalized medicine, including planning targeted therapeutic options and properly scheduling the timing for follow-up appointments and future exams [17]. For instance, patients with *TTN* truncating variants (such as patients 2 and 3) are associated with recovery of LVEF with standard HF treatment [6,17].

In addition, risk stratification for SCD should also be carefully evaluated in all ICMP patients, considering the estimated life expectancy. Identifying a pathogenic variant in any patient with ICMP not only provides clinical prognostic information but also may contribute to the indications for device implantation [6]. However, little information is available about possible additional risk provided by the cardiotoxic drugs in SCD risk stratification. To date, identifying those DCM patients who are at a high arrhythmogenic risk, even in the absence of cardiotoxic therapies still remains a real clinical challenge. In this regard, genetic information can be crucial. For instance, in patient 1, who presented left ventricular dysfunction, a pathogenic truncating variant in *FLNC* was identified. Pathogenic variants in certain DCM genes, like *FLNC*, have been reported in highly arrhythmogenic phenotypes with minimal or subtle structural defects [44]. Therefore, in current ESC guidelines, ICD implantation in primary prevention should be considered in those with a high-risk genetic background for SCD even if their LVEF is higher than 35%. Moreover, in relatives who are carriers of a causative genetic variant associated with increased risk of SCD, like *FLNC*, complete phenotypical evaluation with CMR, even with normal LVEF by TTE, is recommended [17].

Apart from that, if a pathogenic variant is identified in the proband, this information is also very useful for the family. Genetic information enables family counseling and identifying at-risk carriers of the familial pathogenic variant through the proven cost-effective cascade screening [21,45].

## 5. Limitations

We present the results of a single-center study on the experience of breast cancer patients and, consequently, mostly women. We do not believe that general CV risk assessment by Cardio-Oncology Units should be extended to all cancer patients. We strongly believe that CV risk stratification assessment (including family history and ECG) should continue to be performed by the treating oncology or hematology team to identify the patients who really benefit from referring. It is of utmost importance that Cardio-Oncology Units are able to provide specific evaluations without delay, but only in selected patients. If all patients were referred indefinitely, these units would not be able to respond in time to those who really need them. Our approach was only intended for those patients who were already programmed for mandatory basal TTE.

## 6. Conclusions

The 2022 ESC cardio-oncology guidelines recommend a comprehensive baseline assessment of CV toxicity risk before cardiotoxic therapies. Opportunistic screening of concealed ICMPs using clinical history, ECG and TTE provides important information to re-stratify the baseline CV toxicity risk and to optimize preventive strategies and cancer treatment monitoring. It also allows for cascade family testing and the extension of preventive strategies to all available relatives with concealed high cardiovascular risk.

## Figures and Tables

**Table 1:** Clinical characteristics of breast cancer patients evaluated at the Cardio-Oncology Unit from 2020 to 2022.

	Breast Cancer Patients (*n* = 591)
HER2 positive	36.04% (213)
Cancer stage IV	16.75% (99)
Family history of cardiovascular disease	
None	94.25% (557)
Sudden death *	1.52% (9)
Cardiomyopathies	0.68% (4)
Premature coronary artery disease	1.52% (9)
Others	0.17% (1)
Second-degree relatives or at an older age	1.69% (10)
Personal history of cardiovascular disease	
None	93.57% (553)
Arrythmias	2.54% (15)
Coronary artery disease	1.86% (11)
Valvular heart diseases	0.68% (4)
Prior cardiac dysfunction due to chemotherapy	1.18% (7)
Cardiovascular risk factors	
Tabaco consumption	47.04% (278)
Hypertension	24.03% (142)
Diabetes	7.78% (46)
Dyslipidemia	25.55% (151)
Body mass index (kg/m^2^)	27.4 (±6.1)
Mean of available echocardiogram parameters ^†^	Basal/Final
LVMWT (mm)	1 ± 0.2/1 ± 0.2
LVTDV (mL)	46.7 ± 19.4/1 ± 0.2
LVEF	62 ± 6/61.45 ± 5.8
E/A	1 ± 0.6/1 ± 0.4
E/e’	7.2 ± 2.75/7.2 ± 2.8
GLS ^†^	−20% ± 3.4/−20% ± 0.4
% of patients with significant LVEF reduction in follow-up	3.7% (22) 7.2% in HER2 positive vs. 1.3% in HER2 negative (*p* < 0.001)
TAPSE	2.3 ± 0.5/2.23 ± 0.3

* Sudden death was considered positive in first-degree relatives and those < 60 years old, to include inherited cardiomyopathies and premature coronary artery disease. LVMWT: left ventricular maximum wall thickness; LVTDV: left ventricular tele-diastolic volume; LVEF: left ventricular ejection fraction (mm); ^†^ LVEF evaluation by 3D was unavailable and GLS only in some patients.

**Table 2:** Clinical characteristics and genetic variants identified by NGS gene sequencing in the 8 patients with final definitive diagnosis of inherited cardiomyopathy.

Patient	Cardiomyopathy	Genetic Results	Birth Date	ECG	LVEF
1	NDLVC (reclassified from ACM)	*FLNC* c.82060_82061del (p.Tyr1042Ter)	11 May 1975	Abnormal	48%
2	DCM	*TTN* c.82060_82061del (p.Lys27354ValfsTer7)	23 June 1962	Abnormal	Fluctuant
3	DCM	*TTN* c.28074 + 1G > T (IVS112 + 1G > T)	13 May 1963	Normal	45%
4	DCM	Negative	10 January 1963	Abnormal	35%
5	HCM	Negative	20 September 1949	Abnormal	>55%
6	HCM	Negative	18 December 1956	Abnormal	>55%
7	Apical HCM	Negative	11 July 1948	Abnormal	>55%
8	NDLVC (reclassified from LVNC)	Negative	10 May 1974	Abnormal	>55%

LVEF: left ventricular ejection fraction; DCM: familial dilated cardiomyopathy; HCM: hypertrophic cardiomyopathy; ACM: arrhythmogenic cardiomyopathy; NDLVC, non-dilated left ventricular cardiomyopathy; LVNC: left ventricular non-compaction cardiomyopathy.

**Table 3:** Inherited cardiomyopathies prevalence comparison [16,19,29,30,31,32,33,34,35].

Prevalence	Breast Cancer Cohort	General Population (Lowest)	*p* Value	Breast Cancer Cohort	*p* Value
**ACM**	0.0017	0.0002	0.010 *	0.0017	0.593
**DCM**	0.0051	0.002	0.094	0.0051	0.676
**HCM**	0.005	0.0002	<0.001 *	0.005	0.099
**LVNC**	0.0017	0.00014	0.011 *	0.0017	0.015

DCM: familial non-ischemic dilated cardiomyopathy; HCM: hypertrophic cardiomyopathy; ACM: arrhythmogenic cardiomyopathy; LVNC: left ventricular non-compaction cardiomyopathy. * *p* < 0.05.

## Data Availability

No new data were created or analyzed in this study. Data sharing is not applicable to this article.

## Supplementary Materials

The following supporting information can be downloaded at: https://www.mdpi.com/article/10.3390/jcm13010002/s1, Table S1: NGS-Cardiovascular gene panel sequenced for all inherited cardiovascular conditions at our institution.
