# Supplementary material for: Concealed Inherited Cardiomyopathies Detected in Cardio-Oncology Screening
Source: J Clin Med. 2023 Dec 19;13(1):2. doi: 10.3390/jcm13010002 (PMC10780282; doi:10.3390/jcm13010002)
Supplement: Supplementary file 1 [file jcm-13-00002-s001.zip › jcm-2727304-supplementary.pdf]

Supplementary Table S1. NGS-Cardiovascular gene panel sequenced for all inherited cardiovascular conditions at our institution.

Gene	Chromosome	Num. Amplicons	TotalBases	Covered Bases	Missed Bases	Overall Coverage (%)
ABCC8	chr11	47	5139	5139	0	100
ABCC9	chr12	44	5178	5178	0	100
ACTA2	chr10	8	1214	1214	0	100
ACTC1	chr15	8	1194	1194	0	100
ACTN2	chr1	28	3112	3112	0	100
ACVRL1	chr12	14	1602	1602	0	100
AKAP9	chr7	90	12224	12224	0	100
ANK2	chr4	93	12411	12411	0	100
ANKRD1	chr10	9	1050	1050	0	100
APOB	chr2	85	13982	13982	0	100
APOE	chr19	11	1232	1232	0	100
APPL1	chr3	34	3230	3230	0	100
B3GAT3	chr11	13	1474	1471	3	99.8
BAG3	chr10	12	1768	1768	0	100
BGN	chrX	12	1457	1457	0	100
BLK	chr8	21	2118	2118	0	100
BMP10	chr2	9	1375	1375	0	100
BMPR1A	chr10	13	1709	1709	0	100
BMPR1B	chr4	13	1709	1709	0	100
BMPR2	chr2	25	3247	3247	0	100
BRAF	chr7	23	2481	2481	0	100
CACNA1C	chr12	64	7626	7626	0	100
CACNA1D	chr3	64	7150	7150	0	100
CACNA2D1	chr7	51	5327	5327	0	100
CACNB2	chr10	26	3269	3269	0	100
CALM1	chr14	7	690	690	0	100
CALM2	chr2	7	664	664	0	100
CALM3	chr19	8	750	750	0	100
CALR3	chr19	14	1605	1574	31	98.07
CASQ2	chr1	13	1750	1750	0	100
CAV1	chr7	6	687	687	0	100
CAV3	chr3	4	476	476	0	100
CBL	chr11	23	2881	2881	0	100
CBS	chr21	21	1806	1806	0	100
CHRM2	chr7	8	1451	1451	0	100
CHST14	chr15	7	1181	1181	0	100
COL1A1	chr17	56	4905	4905	0	100
COL1A2	chr7	55	4621	4621	0	100
COL3A1	chr2	57	4911	4911	0	100
COL5A1	chr9	78	6256	6256	0	100
COL5A2	chr2	64	7200	7200	0	100

CRYAB	chr11	5	678	678	0	100
CSRP3	chr11	6	635	635	0	100
CTNNA3	chr10	29	3758	3758	0	100
CTNNB1	chr3	19	2486	2486	0	100
DES	chr2	15	1503	1503	0	100
DMD	chrX	106	12161	12161	0	100
DNAJC19	chr3	6	411	411	0	100
DOLK	chr9	9	1667	1667	0	100
DSC2	chr18	25	2912	2912	0	100
DSG2	chr18	28	3507	3507	0	100
DSP	chr6	57	8856	8856	0	100
DTNA	chr18	29	3795	3795	0	100
EIF2AK3	chr2	32	4201	4201	0	100
EIF2AK4	chr15	51	6900	6900	0	100
ELN	chr7	37	4094	4094	0	100
EMD	chrX	9	1005	1005	0	100
ENG	chr9	22	2753	2753	0	100
EYA4	chr6	26	3039	3039	0	100
FBN1	chr15	73	9266	9266	0	100
FBN2	chr5	88	9389	9389	0	100
FHL1	chrX	11	1472	1466	6	99.59
FHOD3	chr18	48	6319	6319	0	100
FKRP	chr19	11	1498	1498	0	100
FKTN	chr9	13	1509	1509	0	100
FLNA	chrX	76	10294	10294	0	100
FLNC	chr7	75	8658	8658	0	100
GAA	chr17	27	3049	3049	0	100
GATA4	chr8	12	1392	1392	0	100
GATA5	chr20	12	1494	1494	0	100
GATAD1	chr7	9	1060	1060	0	100
GCK	chr7	17	1608	1608	0	100
GDF2	chr10	7	1390	1390	0	100
GLA	chrX	10	1360	1360	0	100
GLIS3	chr9	22	3293	3293	0	100
GPD1L	chr3	11	1376	1376	0	100
HCN4	chr15	28	4012	4012	0	100
HFE	chr6	9	1125	1107	18	98.4
HNF1A	chr12	16	2017	2017	0	100
HNF1B	chr17	16	1856	1856	0	100
HNF4A	chr20	20	1860	1860	0	100
HRAS	chr11	5	683	683	0	100
ILK	chr11	14	1959	1959	0	100
INS	chr11	3	433	433	0	100
JAG1	chr20	35	3917	3917	0	100
JPH2	chr20	17	2402	2402	0	100

JUP	chr17	21	2368	2368	0	100
KCNA5	chr12	11	1892	1892	0	100
KCND2	chr7	15	2193	2193	0	100
KCND3	chr1	17	2318	2318	0	100
KCNE1	chr21	2	400	400	0	100
KCNE2	chr21	2	382	382	0	100
KCNE3	chr11	2	362	362	0	100
KCNE5	chrX	3	479	479	0	100
KCNH2	chr7	28	4017	4017	0	100
KCNJ11	chr11	6	1183	1183	0	100
KCNJ2	chr17	8	1294	1294	0	100
KCNJ5	chr11	9	1340	1340	0	100
KCNJ8	chr12	8	1375	1375	0	100
KCNK3	chr2	9	1285	1285	0	100
KCNQ1	chr11	20	2206	2206	0	100
KLF11	chr2	11	1739	1739	0	100
KRAS	chr12	6	737	737	0	100
LAMA4	chr6	55	7540	7540	0	100
LAMP2	chrX	15	1626	1626	0	100
LDB3	chr10	24	2668	2664	4	99.85
LDLR	chr19	21	2763	2763	0	100
LDLRAP1	chr1	14	1377	1377	0	100
LIPA	chr10	14	1650	1650	0	100
LMNA	chr1	18	2369	2369	0	100
LOX	chr5	13	1604	1604	0	100
LZTR1	chr22	29	2733	2733	0	100
MAP2K1	chr15	12	1292	1292	0	100
MAP2K2	chr19	13	1313	1313	0	100
MAT2A	chr2	11	1638	1638	0	100
MED12	chrX	59	6984	6984	0	100
MFAP5	chr12	9	972	972	0	100
MIB1	chr18	36	4071	4071	0	100
MURC	chr9	7	1195	1195	0	100
MYBPC3	chr11	35	4155	4155	0	100
MYH11	chr16	51	6391	6391	0	100
MYH6	chr14	51	6190	6190	0	100
MYH7	chr14	45	6188	6188	0	100
MYL2	chr12	7	571	571	0	100
MYL3	chr3	6	648	648	0	100
MYLK	chr3	41	6055	6055	0	100
MYLK2	chr20	21	2391	2391	0	100
MYOZ2	chr4	8	1045	1045	0	100
MYPN	chr10	36	4983	4983	0	100
NEBL	chr10	38	5002	5002	0	100
NEUROD1	chr2	6	1121	1121	0	100

NEXN	chr1	21	2628	2628	0	100
NF1	chr17	74	9161	9161	0	100
NKX2-5	chr5	9	1142	1142	0	100
NNT	chr5	37	4311	4306	5	99.88
NOTCH1	chr9	63	8008	7987	21	99.74
NOTCH3	chr19	51	7296	7296	0	100
NPPA	chr1	4	486	486	0	100
NRAS	chr1	5	610	610	0	100
PAX4	chr7	10	1482	1482	0	100
PAX6	chr11	13	2035	2035	0	100
PCSK9	chr1	16	2199	2199	0	100
PDLIM3	chr4	13	1733	1733	0	100
PDX1	chr13	7	952	952	0	100
PKP2	chr12	21	2786	2786	0	100
PLN	chr6	1	169	169	0	100
PLOD1	chr1	28	3325	3325	0	100
PPARG	chr3	12	1588	1588	0	100
PRDM16	chr1	37	4681	4654	27	99.42
PRKAG2	chr7	19	1974	1974	0	100
PRKG1	chr10	26	3087	3087	0	100
PSEN1	chr14	12	1504	1504	0	100
PSEN2	chr1	15	1447	1447	0	100
PTPN11	chr12	16	1936	1936	0	100
RAF1	chr3	18	2107	2107	0	100
RASA1	chr5	39	3412	3411	1	99.97
RASA2	chr3	38	3815	3793	22	99.42
RBM20	chr10	27	3824	3817	7	99.82
RIT1	chr1	8	771	771	0	100
RYR2	chr1	134	15954	15954	0	100
SCN10A	chr3	49	7221	7221	0	100
SCN1B	chr19	9	1066	1066	0	100
SCN2B	chr11	7	848	848	0	100
SCN3B	chr11	8	898	898	0	100
SCN4B	chr11	7	887	887	0	100
SCN5A	chr3	44	6423	6423	0	100
SDHA	chr5	19	2145	2145	0	100
SGCD	chr5	8	1025	1025	0	100
SHOC2	chr10	14	1829	1829	0	100
SKI	chr1	18	2537	2537	0	100
SLC22A5	chr5	14	1856	1856	0	100
SLC2A10	chr20	14	1876	1876	0	100
SLMAP	chr3	33	3799	3799	0	100
SMAD1	chr4	12	1698	1698	0	100
SMAD2	chr18	13	1504	1504	0	100
SMAD3	chr15	14	1452	1452	0	100

SMAD4	chr18	18	1769	1769	0	100
SMAD9	chr13	13	1704	1704	0	100
SNTA1	chr20	17	1918	1918	0	100
SOS1	chr2	40	4232	4232	0	100
SOS2	chr14	46	4919	4919	0	100
SPRED1	chr15	13	1405	1405	0	100
TAZ	chrX	14	1043	1043	0	100
TBX20	chr7	15	1748	1748	0	100
TBX4	chr17	16	2038	2038	0	100
TBX5	chr12	12	1637	1637	0	100
TCAP	chr17	5	524	524	0	100
TGFB2	chr1	12	1409	1409	0	100
TGFB3	chr14	10	1589	1589	0	100
TGFBR1	chr9	13	1614	1614	0	100
TGFBR2	chr3	12	1859	1859	0	100
TMEM43	chr3	13	1323	1323	0	100
TMPO	chr12	25	3385	3385	0	100
TNNC1	chr3	7	546	546	0	100
TNNI3	chr19	7	702	702	0	100
TNNI3K	chr1	30	3758	3710	48	98.72
TNNT2	chr1	17	1081	1081	0	100
TPM1	chr15	15	1500	1500	0	100
TRDN	chr6	48	4418	4418	0	100
TRPM4	chr19	32	3895	3895	0	100
TTN	chr2	800	132581	129775	2806	97.88
TTR	chr18	5	484	484	0	100
TXNRD2	chr22	23	2543	2543	0	100
VCL	chr10	36	4505	4505	0	100
ZDHHC9	chrX	10	1545	1545	0	100
